# Fine-Tuning Bi_2_Te_3_-Copper Selenide Alloys Enables an Efficient n-Type Thermoelectric Conversion

**DOI:** 10.3390/molecules27238183

**Published:** 2022-11-24

**Authors:** Longbin Li, Jianchao Jia, Chaosheng Shi, Wei Zeng

**Affiliations:** Flexible Sensing Technology Research Center, Guangdong Provincial Key Laboratory of Industrial Surfactant, Institute of Chemical Engineering, Guangdong Academy of Sciences, Guangzhou 510665, China

**Keywords:** bismuth telluride, copper selenide, alloy, n-type thermoelectric material

## Abstract

Bismuth tellurides is one of the most promising thermoelectric (TE) material candidates in low-temperature application circumstances, but the n-type thermoelectric property is relatively low compared to the p-type counterpart and still needs to be improved. Herein, we incorporated different copper selenides (CuSe, Cu_3_Se_2_ and Cu_2−x_Se) into a Bi_2_Te_3_ matrix to create the alloy by grinding and successive sintering to enable higher thermoelectric performance. The results demonstrated that all alloys achieved n-type TE characteristics and Bi_2_Te_3_-CuSe exhibited the best Seebeck coefficient and power factor among them. Along with the low thermal conductivity, the maximum dimensionless TE figure of merit (ZT) value of 1.64 at 573 K was delivered for Bi_2_Te_3_-CuSe alloy, which is among the best reported results in the n-type Bi_2_Te_3_-based TE materials to the best of our knowledge. The improved TE properties should be related to the co-doping process of Se and Cu. Our investigation shows a new method to enhance the performance of n-type TE materials by appropriate co-doping or alloying.

## 1. Introduction

As both the practical need and the worldwide market for wearable and implantable electronics are increasing rapidly [1,2], flexible thermoelectric (FTE) materials, which are fundamental in the manufacturing of such devices, have been receiving particular attention in recent years. To maximize the electro-thermal conversion efficiency and reachability [3], the FTE materials should exhibit satisfactory thermoelectric properties at a relatively low temperature range [4,5]. Among all kinds of TE materials, bismuth telluride-based materials are considered some the most attractive and promising FTE materials for both p-type and n-type TE applications to date [6,7,8,9]. The advantages of bismuth telluride-based materials include its highest ZT value and stable performance around room temperature, ease of preparation and moderate mechanical properties [5,10]. Until now, the ZT values of p-type bismuth telluride-based materials are usually reported higher than those of the n-type counterpart [11,12,13,14,15,16,17,18,19,20]. For instance, the highest ZT value of p-type bismuth telluride-based materials has reached about 1.86 [13], whilst the highest value is merely 1.47 for the n-type counterpart and most of other reported high ZT values are around 1.2 [18,19,20]. For the sake of maximizing the energy conversion efficiency of a practical TE device, the ZT value of the p-type and n-type materials should be equally high and close to each other [21]. Thus, the TE performance of n-type bismuth telluride-based materials still needs to be further improved.

In recent years, nano-structuring and alloying have been shown to be the simple and effective strategies to prompt TE properties [7,22]. For instance, Lou et al. discovered that Na-doping can influence the Fermi level properly, and thereby optimizing the electrical and thermal transport properties of Bi_2_Te_3_ [23]. Therefore, the peak ZT value of the doped sample attained a 70% enhancement compared to that of the pristine sample. Zhu et al. also boosted the ZT value of n-type Bi_2_Te_3_ to 1.35 by W and Cl co-doping [24]. They revealed that co-doping can optimize the carrier concentration and enlarge van der Waals gap for better scattering of phonons. These reports demonstrated that proper alloying or co-doping of Bi_2_Te_3_ can boost TE performance by band tuning or promoting the boundary scattering of phonons. However, it is still unclear how to wisely choose the suitable doping/alloying components to enhance the properties of Bi_2_Te_3_, and there is still a relatively large amount of room for a more favorable n-type TE performance by trail-and-error.

Similar to bismuth tellurides, copper selenides are another candidate of promising TE materials. It was revealed that the β-phase of Cu_2−x_Se exhibits the ideal phonon-glass electron-crystal model, and is also relatively economical and eco-friendly [25,26,27,28]. More importantly, the very high ZT value, up to 2.3~2.7, of Cu_2_Se-based materials was demonstrated as a characteristic of the p-type TE, due to the optimal carrier concentration and intensive phonon scattering [29,30]. Some researchers have revealed that alloying with Se or some metallic cations can easily affect the conduction band structure, which is effective to tune the carrier concentration and transport property [23,31,32,33,34]. When copper selenides is incorporated into the Bi_2_Te_3_ matrix, it might very probably realize a better n-type TE performance.

In this work, we synthesized Bi_2_Te_3_-based alloys with copper selenides (CuSe, Cu_3_Se_2_ and Cu_2−x_Se) and studied the effects of adding copper selenides. Our results revealed that three alloys showed a negative Seebeck coefficient characteristic, and the Bi_2_Te_3_-CuSe alloy exhibited the highest absolute value, over 200 μV/K around 433 K, among them. Moreover, the electrical conductivity of Bi_2_Te_3_-CuSe is higher than the reference Bi_2_Te_3_. The thermal conductivity remained relatively low and displayed a decreasing trend with the temperature rising from 303 to 573 K. Therefore, the synergistic effect of three parameters resulted in the highest ZT values, up to 1.64 at 573 K for the Bi_2_Te_3_-CuSe alloy, which is remarkable for the n-type TE materials. Our results demonstrated a novel method for enhancing the ZT value of efficient n-type TE materials.

## 2. Experimental Section

Fabrication of bismuth telluride-copper selenide alloys: Bi_2_Te_3_ powders (Macklin, 99.9%) were purchased commercially. The copper selenides with different stoichiometric compositions (CuSe, Cu_3_Se_2_ and Cu_2−x_Se) were prepared by wet chemical methods, which can be seen in our previous article [25]. Bi_2_Te_3_ and copper selenides powders were mixed in an agate mortar, and then fully ground for about 20 min. The molar ratio of Bi_2_Te_3_ and different copper selenides were all set as 1:0.62. Additionally, samples with different molar ratios of Bi_2_Te_3_ versus CuSe (1:1~1:0.2) were also prepared to investigate the best alloyed proportion. The evenly mixed powders were transferred into a cylindrical mold and pressed under 30 MPa pressure which lasted for 10 min. After that, the samples turned into hard disks with diameters of about 1.3 cm and were successively sintered in a tube furnace at 573 K for 2 h under flowing Ar stream.

Characterization of samples: The crystal structures of the samples were measured by X-ray diffractometry (XRD, Ultima IV, Rigaku Corporation, Tokyo, Japan). Further structural and phase identification were examined by transmission electron microscopy and high-resolution transmission electron microscopy (TEM and HRTEM, Talos F200x, FEI, Waltham, MA, USA), and elemental mapping were conducted under TEM characterization.

Seebeck coefficient and electrical conductivity of the samples were both tested by Seebeck coefficient measurement (LSR-3, Linseis, Selb, Germany) at the range between 303 to 573 K, which was conducted in He atmosphere. Thermal conductivity was directly measured by thermal conductivity analyzer (TPS 2500S, Hot Disk, Göteborg, Sweden) at the temperature range of 333 to 573 K.

## 3. Results and Discussion

**Figure 1:**
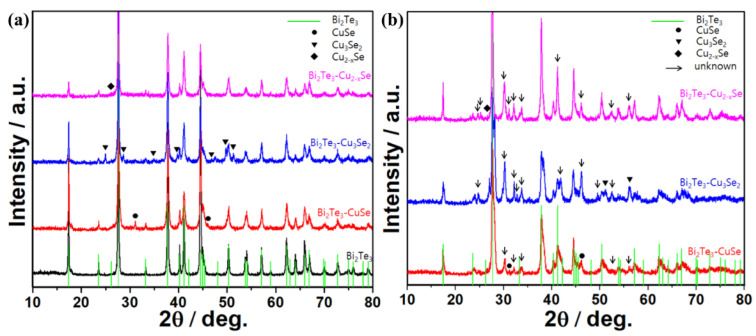
(**a**) XRD patterns of pristine Bi_2_Te_3_ and the composites without calcination. (**b**) XRD patterns of Bi_2_Te_3_-based alloys with calcination.

The crystal structures of Bi_2_Te_3_-copper selenides alloys with/without calcination were characterized using XRD method, where the commercial Bi_2_Te_3_ was used as reference. As shown in Figure 1a, one can notice that the untreated Bi_2_Te_3_ displays a typical XRD pattern of mineral tellurobismuthite Bi_2_Te_3_ (JCPSD Card No. 15-0863), and the blended composite without calcination shows superposed patterns of Bi_2_Te_3_ and the corresponding copper selenides. For example, the diffraction peaks at 31.1° and 46.0° of the Bi_2_Te_3_-CuSe composite belong to the klockmannite CuSe (JCPDS Card No. 34-0171), and the peaks at 25.0°, 28.7°, etc., of the Bi_2_Te_3_-Cu_3_Se_2_ composite belong to the umangite Cu_3_Se_2_ (JCPDS Card No. 47-1745), and the peak at 26.7° of the Bi_2_Te_3_-Cu_2−x_Se composite belongs to the berzelianite Cu_2−x_Se (JCPDS Card No. 06-0680). However, the XRD patterns of the alloys with calcination treatment turned out to be very different, as displayed in Figure 1b. Several new peaks appear in the patterns and they are hard to classify. Such peaks possibly reflect the existence of non-stoichiometric compounds composed of Bi, Te, Cu and Se, which should have an impact on their TE properties.

**Figure 2:**
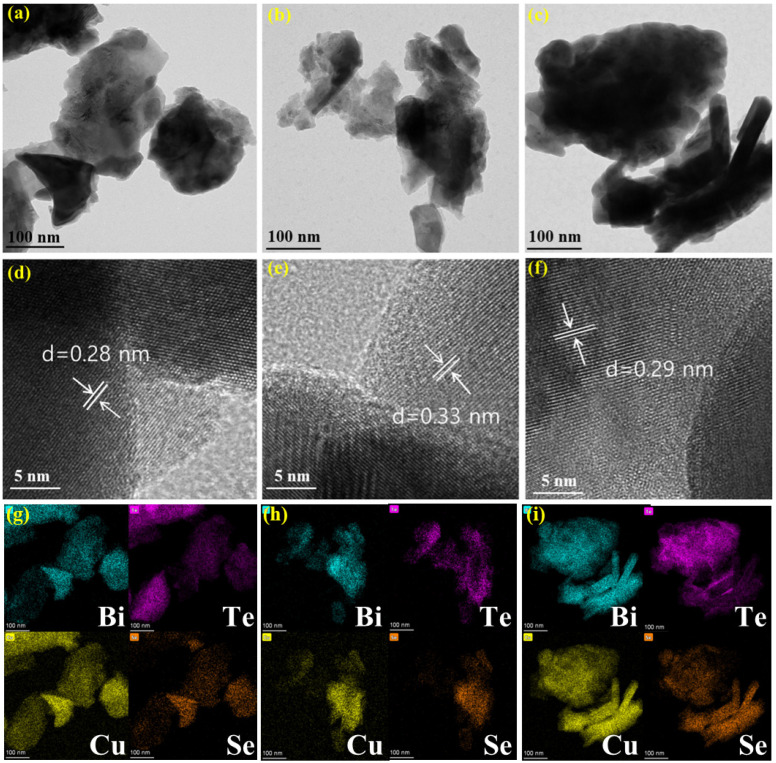
TEM (**a**–**c**), HRTEM (**d**–**f**) and corresponding elemental mapping (**g**–**i**) images of the Bi_2_Te_3_-CuSe, Bi_2_Te_3_-Cu_3_Se_2_ and Bi_2_Te_3_-Cu_2−x_Se alloys, respectively. Bi, Te, Cu and Se elements are marked by blue, magenta, yellow and orange in (**g**–**i**), respectively.

The sintered alloys were crushed into powders and then examined with TEM, HR-TEM and elemental mapping characterizations, and the relevant images are shown in Figure 2. All the alloys are irregular fragments with bulks with sizes of hundreds of nanometers, and no specific characteristics can be recognized in TEM images. The interplanar space distances of the alloys estimated from HRTEM images are 0.28 nm, 0.33 nm and 0.29 nm for Bi_2_Te_3_-CuSe, Bi_2_Te_3_-Cu_3_Se_2_ and Bi_2_Te_3_-Cu_2−x_Se_2_, respectively. Such values are close to the lattice spacing (0.32 nm) of (015) of Bi_2_Te_3_, but do not match well with any other typical crystalline data of Bi_2_Te_3_ or copper selenides. This may result from the co-doping of Se and Cu which changes the crystalline structure of Bi_2_Te_3_. In the elemental mapping images shown in Figure 2g–i, it can be observed that four elements of Bi, Te, Cu and Se are all evenly distributed throughout the alloy bulk, indicating that copper selenides fused well with the Bi_2_Te_3_ substrate. Such result can also be seen in the SEM and EDS mapping images which are shown in the Supplementary Materials (Figures S1 and S2).

**Figure 3:**
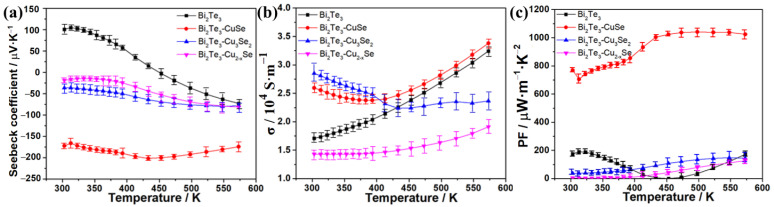
Temperature dependence of Seebeck coefficient (**a**), electrical conductivity (**b**) and power factor (**c**) of pristine Bi_2_Te_3_ and Bi_2_Te_3_-copper selenide-based alloys.

We further comparatively studied the effect of different copper selenide on TE properties of the sintered alloys, which are shown in Figure 3. Figure 3a shows the dependence of their Seebeck coefficients (*S*) on temperature. It can be seen that commercial Bi_2_Te_3_ exhibits the p-type TE characteristic between 303~453 K, but the Seebeck coefficient turns negative at the temperature range of 453~573 K, which is mainly as a result of the sensitivity to particle size, surface defects and manufacturing processes [35,36]. In the cases of the synthesized Bi_2_Te_3_-copper selenide alloys, all of them maintain the n-type characteristic in the entire testing temperature range, and Bi_2_Te_3_-Cu_3_Se_2_ and Bi_2_Te_3_-Cu_2−x_Se alloys have relatively lower absolute *S* values around 15~80 μV·K^−1^. Nevertheless, the absolute S value of the Bi_2_Te_3_-CuSe alloy first rises at 313~433 K and then slightly decreases over 433 K, resulting in an oblate peak value of 201.8 μV K^−1^ at about 433 K. The very high *S* value of Bi_2_Te_3_-CuSe is 2~12 times larger than that of other alloys at the same temperature. The absolute *S* value of Bi_2_Te_3_-CuSe is comparable to the best results reported in other n-type Bi_2_Te_3_-based TE materials [18,19,20,21,23], which is about 170~240 μV/K at the temperature range of room temperature to 575 K. Some recent reports disclosed that both the doped Se and metal atoms can tune the Fermi level and the band structure of Bi_2_Te_3_, which can greatly impact the TE performance [6,23,34]. The measured carrier concentration and calculated effective mass results also indicate the excellent *S* value of Bi_2_Te_3_-CuSe, which is discussed in the Supplementary Materials (Table S1). Thus, it is reasonable that the higher Seebeck coefficient of Bi_2_Te_3_-CuSe alloy is speculated to be related to the appropriate doping of Se and Cu into the Bi_2_Te_3_ crystallites to form an alloy structure (as shown in Figure 1 and Figure 2).

The curves of electrical conductivity (σ)-temperature are shown in Figure 3b. Both Bi_2_Te_3_-CuSe and Bi_2_Te_3_-Cu_3_Se_2_ display comparably high electrical conductivity in the range of 303~413 K, but the latter decreases to be lower than Bi_2_Te_3_ when the temperature increases over 413 K. Conversely, the Bi_2_Te_3_-CuSe alloy maintains the continuously increasing trend after crossing the lowest point at 393 K, which is similar to that of reference Bi_2_Te_3_, and the corresponding σ value remains at the highest levels in the 413~573 K. Bi_2_Te_3_-Cu_2−x_Se shows only a sluggish increase with the rise of temperature, and the σ value is less than 2.0 × 10^4^ S m^−1^.

**Figure 4:**
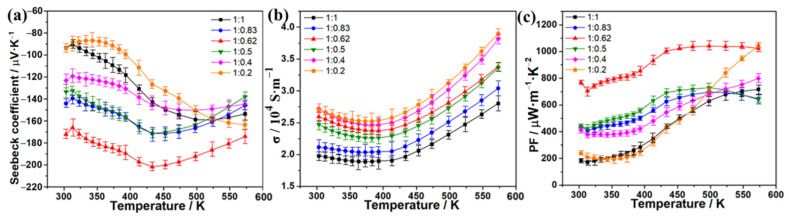
Temperature dependence of Seebeck coefficient (**a**), electrical conductivity (**b**), and power factor (**c**) of the Bi_2_Te_3_-CuSe alloys with different proportions.

**Figure 5:**
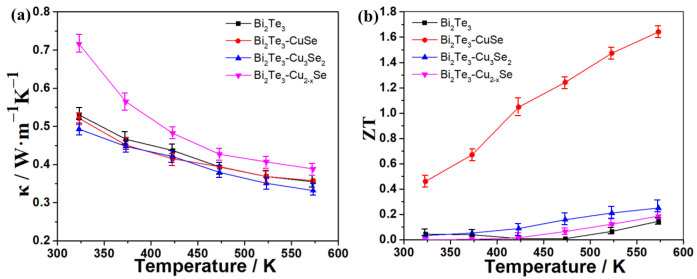
Temperature dependence of thermal conductivity (**a**) and ZT value (**b**) of Bi_2_Te_3_ and the alloys.

Furthermore, the power factors (PF) of these alloys and the reference Bi_2_Te_3_ were calculated from the equation of PF = *S*^2^σ, and the PF–temperature curves are shown in Figure 3c. As expected, the Bi_2_Te_3_-CuSe alloy exhibits the strongest power factors because of the excellent Seebeck coefficient and electrical conductivity. In particular, the highest PF value reached over 1000 μW·m^−1^·K^−2^ in the temperature range of 433~573 K, which is over six times as large as the other alloys and the pristine Bi_2_Te_3_. The PF in the 300~433 K range is slightly lower than the plateau values, mainly resulting from the slightly decreased electrical conductivity. We also fine-tuned the CuSe component in the corresponding alloy materials to further improve the TE properties. Figure 4a shows the Seebeck coefficient results, and the 1:0.62 sample exhibits the highest absolute Seebeck coefficient values throughout the tested temperature range. Figure 4b shows the electrical conductivity result. Overall, when the amount of CuSe increases, the electrical conductivity increases, except that the 1:0.62 sample is slightly higher than the 1:0.5 sample. The calculated PF result is shown in Figure 4c. Thanks to the highest absolute Seebeck coefficient values and the relatively high electrical conductivity, the 1:0.62 sample also demonstrates the highest PF in most of the tested temperature ranges. Furthermore, the thermal conductivity (κ) at different temperatures was determined, and the resulting ZT values are estimated from the equation of ZT = *S*^2^σT/κ, which is shown in Figure 5. The Bi_2_Te_3_-CuSe and Bi_2_Te_3_-Cu_3_Se_2_ alloys exhibit similar thermal conductivity to the reference materials Bi_2_Te_3_ in the entire temperature range, while the value is shown to be largest for the Bi_2_Te_3_-Cu_2−x_Se alloy. However, all the three alloys exhibit lower lattice thermal conductivity and better phonon scattering than the pristine Bi_2_Te_3_, which can be seen in the Supplementary Materials (Figure S3). Therefore, the Bi_2_Te_3_-CuSe alloy possesses the highest ZT values in the tested temperature ranges. It is worth noting that, although the ZT value of 0.46 at 323 K is moderate compared to many other reported near-room-temperature n-type Bi_2_Te_3_-based TE materials, it can be almost linearly augmented to a remarkable level of 1.64 when the temperature rises to 573 K. In fact, the trend is different from the results reported in other studies in the literature [19,20,21,23,37]. This should be ascribed to the fact that the addition of a certain amount of Se may enlarge the bandgap and tune the carrier concentration, thus moving the peak ZT value to a higher temperature, as explained by Pan et al. [38].

## 4. Conclusions

In summary, the Bi_2_Te_3_-based alloys with various copper selenides were prepared by grinding and successive sintering for an efficient thermoelectric conversion. It was found that all of the synthesized alloys exhibited the n-type TE characteristic. Among them, Bi_2_Te_3_-CuSe attained the highest Seebeck coefficient throughout 303~573 K. Additionally, the increasing electrical conductivity and decreasing thermal conductivity led to its peak ZT value reaching 1.64 at 573 K, and such a result is remarkable in n-type Bi_2_Te_3_-based TE materials. The improved TE properties should be related to the alloying structure of Bi_2_Te_3_ and CuSe. Our results demonstrated an efficient n-type TE material with a high ZT value with future practical thermoelectric applications.

## Data Availability

Not applicable.

## Supplementary Materials

The following supporting information can be downloaded at: https://www.mdpi.com/article/10.3390/molecules27238183/s1, Figure S1: SEM and EDS mapping images of the crushed powders of the alloyed samples; Figure S2: Surface SEM and EDS mapping images of the bulks of the alloyed samples; Table S1: Hall carrier concentration (*n_H_*) and Hall carrier mobility (*μ_H_*) of the samples measured at 303 K, and the calculated m^*^ results; Figure S3: the calculated electronic thermal conductivity (к_e_) and the lattice thermal conductivity (к_l_) results of the samples.
