# Supplementary material for: Fine-Tuning Bi2Te3-Copper Selenide Alloys Enables an Efficient n-Type Thermoelectric Conversion
Source: Molecules. 2022 Nov 24;27(23):8183. doi: 10.3390/molecules27238183 (PMC9737956; doi:10.3390/molecules27238183)
Supplement: Supplementary file 1 [file molecules-27-08183-s001.zip › molecules-2025982-supplementary.pdf]

Supporting Information

Fine-tuning Bi_2Te_3-copper selenide alloys enables an efficient n-type thermoelectric conversion

Longbin Li [1], Jianchao Jia [1], Chaosheng Shi [1], and Wei Zeng [1,*]

The scanning electron microscopy (SEM, MIRA LMS, Tescan, Brno, Czech) and elemental mapping images of the alloys are shown as below:

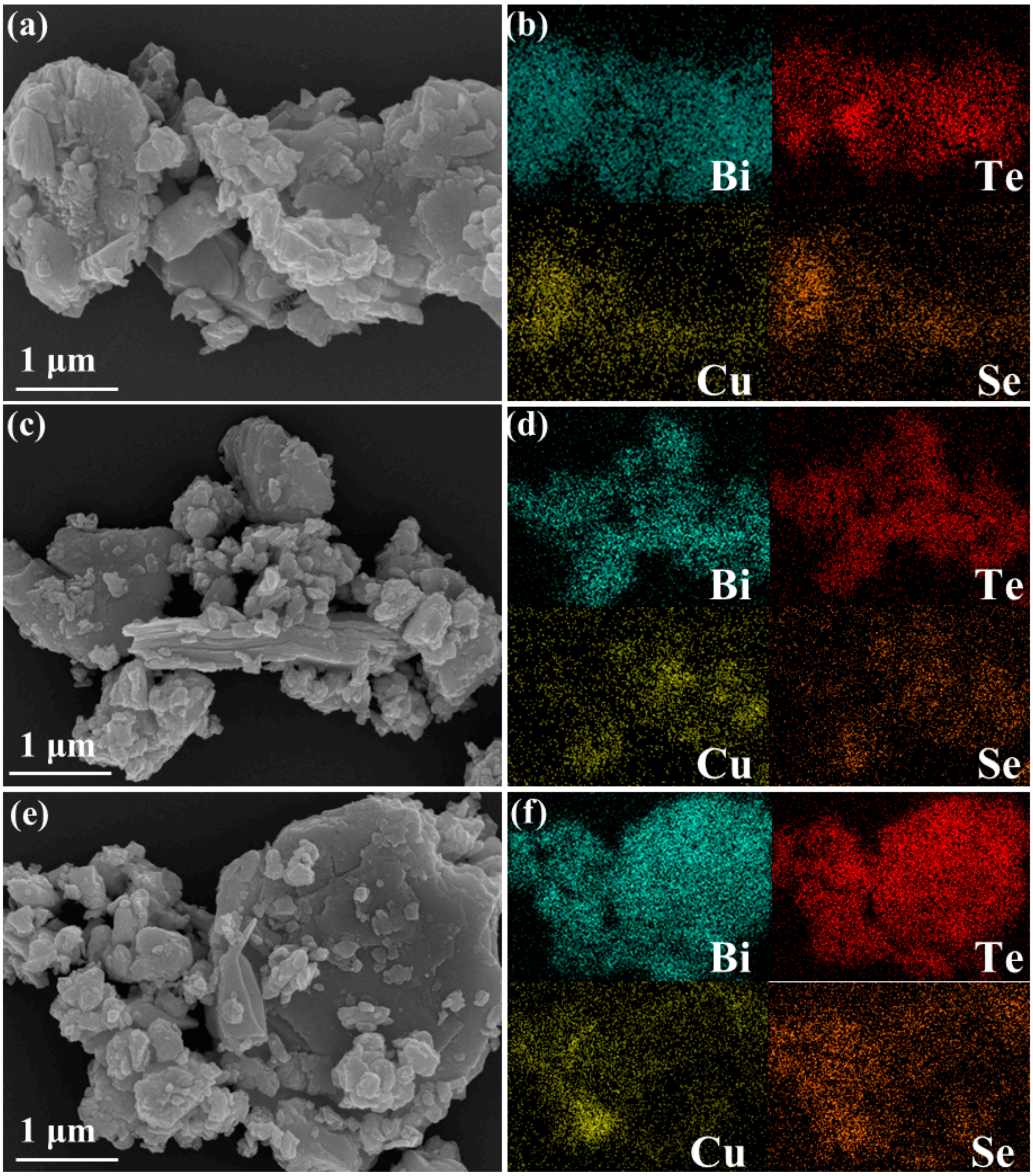

Figure S1 SEM and EDS mapping images of the crushed powders of (a,b)

Bi_2Te_3-CuSe, (c,d) Bi_2Te_3-Cu_3Se_2, and (e,f) Bi_2Te_3-Cu_{2-x}Se, respectively. Bi, Te, Cu and Se elements are marked by blue, magenta, yellow and orange in the mapping images, respectively.

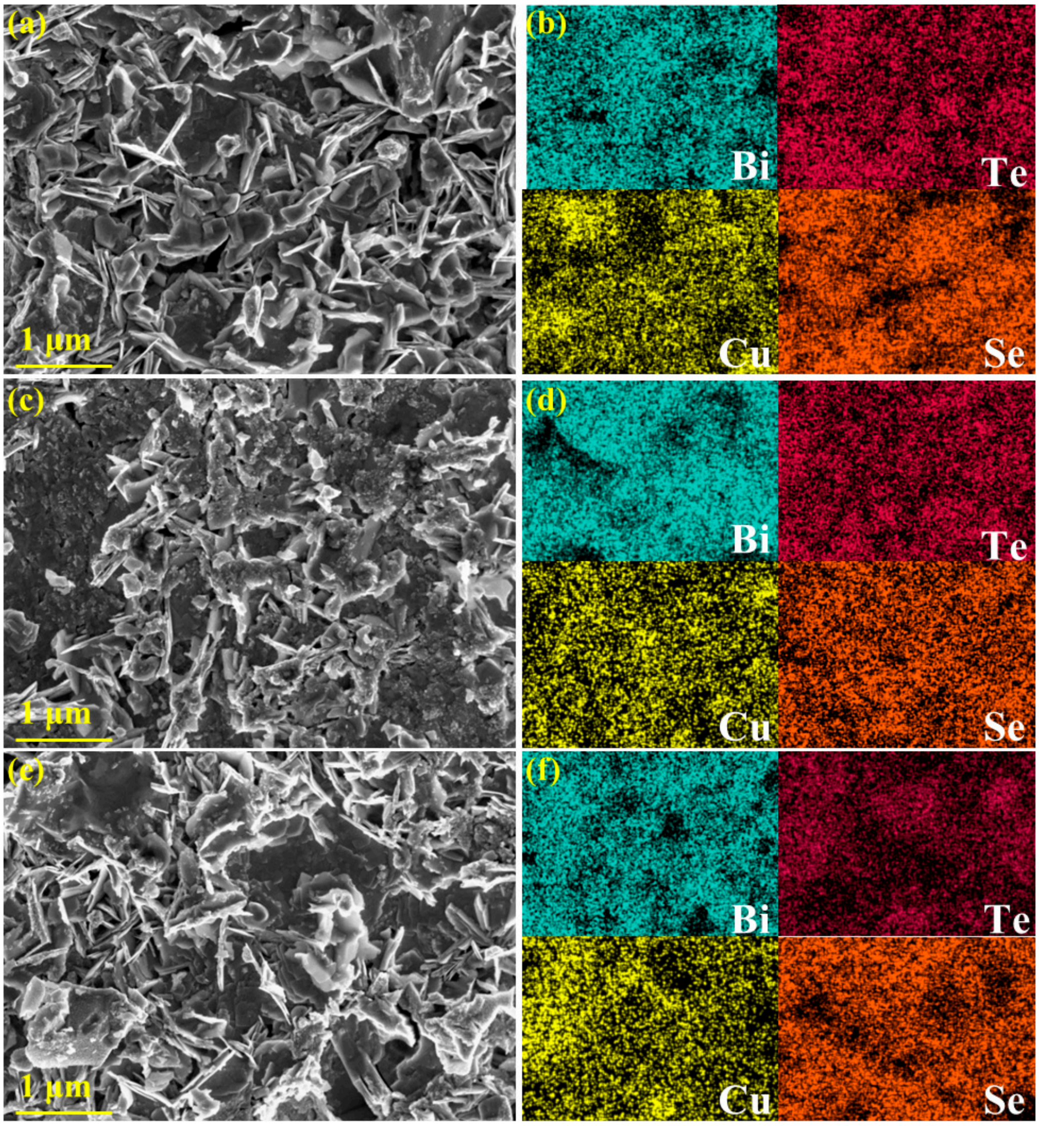

Figure S2 Surface SEM and EDS mapping images of the bulks of the (a,b) Bi_2Te_3-CuSe, (c,d) Bi_2Te_3-Cu_3Se_2, and (e,f) Bi_2Te_3-Cu_{2-x}Se, respectively. Bi, Te, Cu and Se elements are marked by blue, magenta, yellow and orange in the EDS mapping images, respectively.

Table S1 Hall carrier concentration (n_H) and Hall carrier mobility (μ_H) of the samples measured at 303 K, and the calculated m^* results.

Samples	n_H(10^{19} cm^{-3})	μ_H (cm^2 V^{-1} s^{-1})	m^*(m_0)
Bi_2Te_3	3.34	27.44	0.71
Bi_2Te_3-CuSe	5.83	37.75	0.93
Bi_2Te_3-Cu_3Se_2	18.81	14.27	0.74
Bi_2Te_3-$Cu_{2-x}Se$	17.01	6.08	0.51

The Hall carrier concentration (n_H) and Hall carrier mobility (μ_H) of the samples at 303 K by a Hall effect measurement (HMS-5300, ECOPIA, Anyang, Korea), and the effective masses (m^*) are calculated using the following equation:

$$S = \frac{8\pi^2 k_B^2}{3eh^2}\left(\frac{\pi}{3n_H}\right)^{2/3} m^* T$$

where S, k_B, e and h are the absolute Seebeck coefficient value, Boltzmann constant, elementary charge and Planck's constant, respectively.

It is obvious that the Hall carrier concentration of Bi_2Te_3-CuSe is much smaller than that of Bi_2Te_3-Cu_3Se_2 and Bi_2Te_3-$Cu_{2-x}Se$, which contributes to the high absolute Seebeck coefficient value of Bi_2Te_3-CuSe. Additionally, Bi_2Te_3-CuSe exhibits higher effective mass than the other samples. The enhanced effective mass can be explained by the proper band structure change after Cu and Se co-doping. Ref. 23 has disclosed that, the movement of Fermi energy into the conduction band and the increase of total density of states can enhance the effective mass. Thus, the appropriate carrier concentration and band structure change after Cu and Se co-doping should be attributed to the excellent Seebeck coefficient of Bi_2Te_3-CuSe.

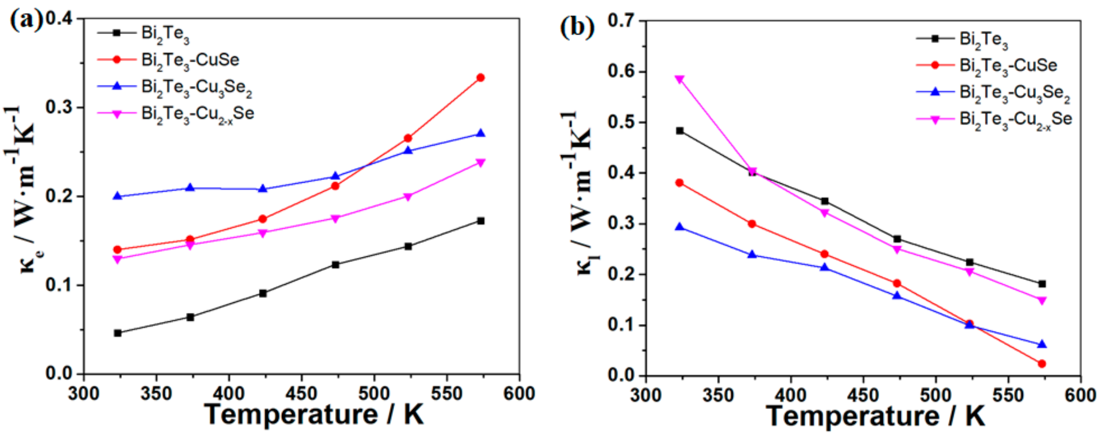

Figure S3 the calculated κ_e (a) and κ_l (b) results of the samples.

The electronic thermal conductivity (κ_e) and the lattice thermal conductivity (κ_l) have been calculated to investigate the phonon scattering. According to the Wiedemann–Frantz law, the following equations were utilized:

$$\kappa_e = L\sigma T$$

where L, σ and T are Lorenz number, electrical conductivity and temperature, respectively. The Lorenz number can be calculated from the equation below:

$$L = 1.5 + \exp\left(-\frac{|S|}{116}\right)$$

where L is in $10^{-8} W\Omega K^{-2}$ and S is Seebeck coefficient and in μV/K.

$$\kappa_l = \kappa - \kappa_e$$

Where κ is the total thermal conductivity that we have measured in the thermal conductivity test. The κ_e and κ_l results are shown as below:

From Figure S3 (b), both Bi_2Te_3-CuSe and Bi_2Te_3-Cu_3Se_2 exhibit lower κ_l than the pristine Bi_2Te_3 in the whole testing temperature range, and Bi_2Te_3-$Cu_{2-x}Se$ also shown lower κ_l than Bi_2Te_3 in the temperature range higher than 373 K, which indicating better phonon scattering in the samples. The improvement of phonon scattering can be ascribed to the lattice distortions which formed in the process of alloying two different compounds.
